# A Review on Ylang-Ylang (*Cananga odorata*) Essential Oil, Its Applications, and Extraction Methods

**DOI:** 10.3390/molecules31183146

**Published:** 2026-09-08

**Authors:** Rasool Shabanloo, Aleksandra Maria Nowak, Dawid Stawski, Somaye Akbari

**Affiliations:** 1 Textile Institutte, Lodz University of Technology, Żeromskiego 116, 90-924 Lódź, Poland; rasool.shabanloo@dokt.p.lodz.pl (R.S.);; 2 Department of Textile Engineering, School of Materials and Advanced Processes Engineering, Amirkabir University of Technology (Tehran Polytechnic), 424 Hafez Ave, Tehran 15875-441, Iran; akbari_s@aut.ac.ir

**Keywords:** *Cananga odorata*, Ylang-Ylang, insect repellent textiles, microencapsulation, bio-finishing, mosquito control, smart textiles

## Abstract

This review provides a comprehensive analysis of Ylang-Ylang (*Cananga odorata*) essential oil (YYEO), describing its botany, historical evolution, and global commercial significance. It systematically provides information on traditional extraction techniques such as hydrodistillation, steam distillation, and solvent extraction alongside innovative green technologies, including microwave-assisted distillation (MAD), supercritical fluid extraction (SFE), and ultrasound-assisted extraction (UAE). Conventional distillation methods are compared with greener technologies. The reviewed studies indicate that microwave-assisted processing can reduce YYEO extraction time from approximately 19 h for conventional hydrodistillation to about 40 min while improving the retention of light oxygenated compounds. In particular, light oxygenated compounds have been reported at approximately 81.23% in solvent-free microwave extracts, compared with 69.94% for hydrodistillation and 57.98% for steam distillation. It has also been reported that YYEO contains more than 50 volatile secondary metabolites, with linalool representing about 28% of the oxygenated fraction, while sesquiterpene-rich hydrocarbons can account for up to 63% of the essential oil. The reviewed studies further demonstrate insecticidal, antimicrobial, antioxidant, anti-inflammatory, and neurobiological activities, supporting the potential use of YYEO in sustainable protective materials and health-related applications. Finally, emerging frontiers in protective smart textiles, living fabrics, and sustainable closed-loop manufacturing paradigms are discussed to outline future directions for bio-based material science.

## 1. Introduction

### Botany and Morphological Characteristics of Cananga odorata and Its History

**Figure 1:**
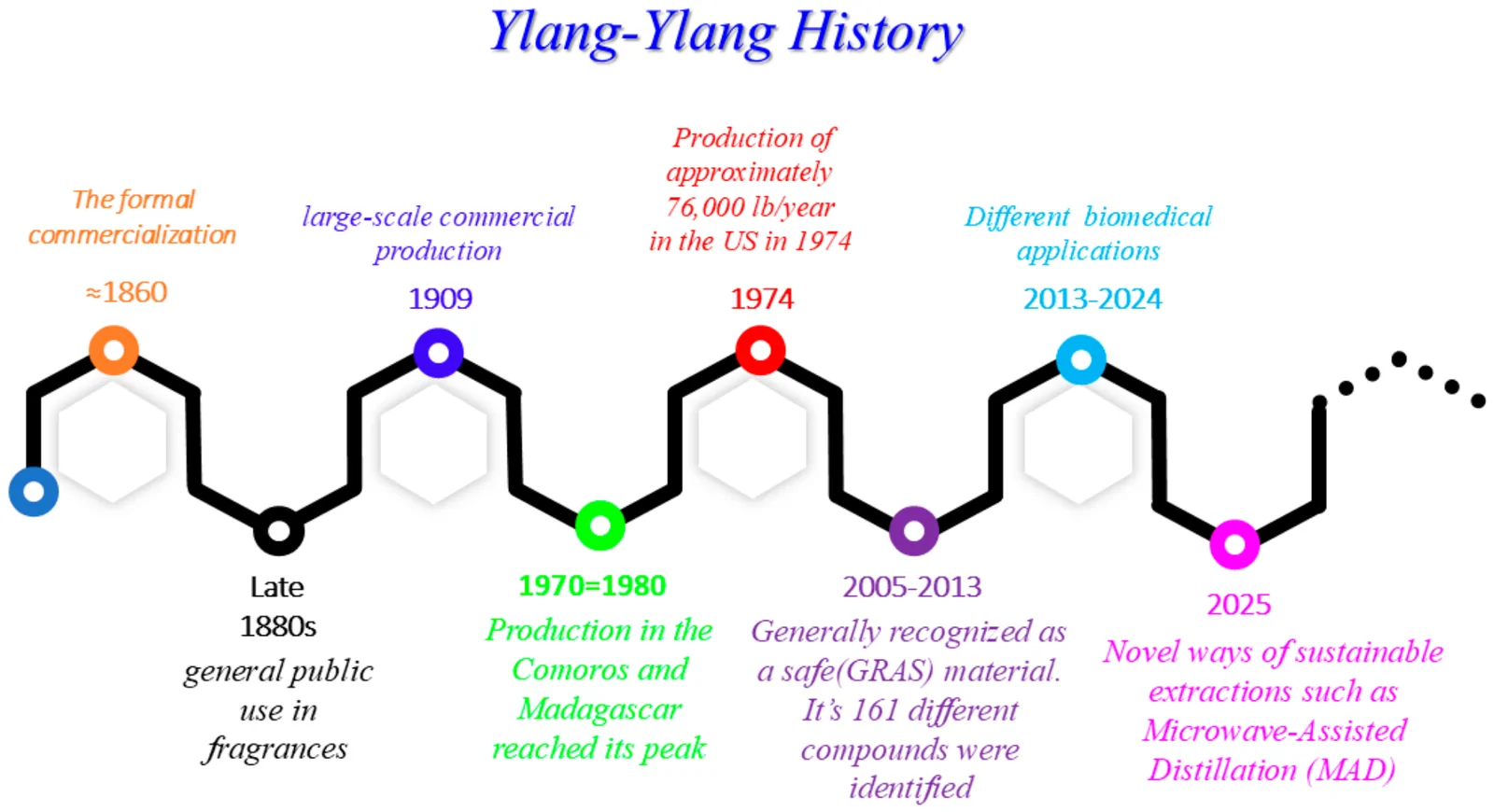
Brief history of YYEO.

*Cananga odorata* (Lam.) Hook.f. & Thomson (*C. odorata*), which is known as Ylang-Ylang, is a fast-growing leaf-retaining tree that is closely related to the custard-apple family, Annonaceae, within the older group of flowering plants, order Magnoliales. The history of the *C. odorata* began in Manila, the Philippines, around 1860, as shown in Figure 1, when Albert Schwenger, a stranded sailor, became enriched by the flower’s scent, leading him to distill a small quantity of oil from a portable wheel cart, where the first commercialization of essential oils began [1,2,3].

**Figure 2:**
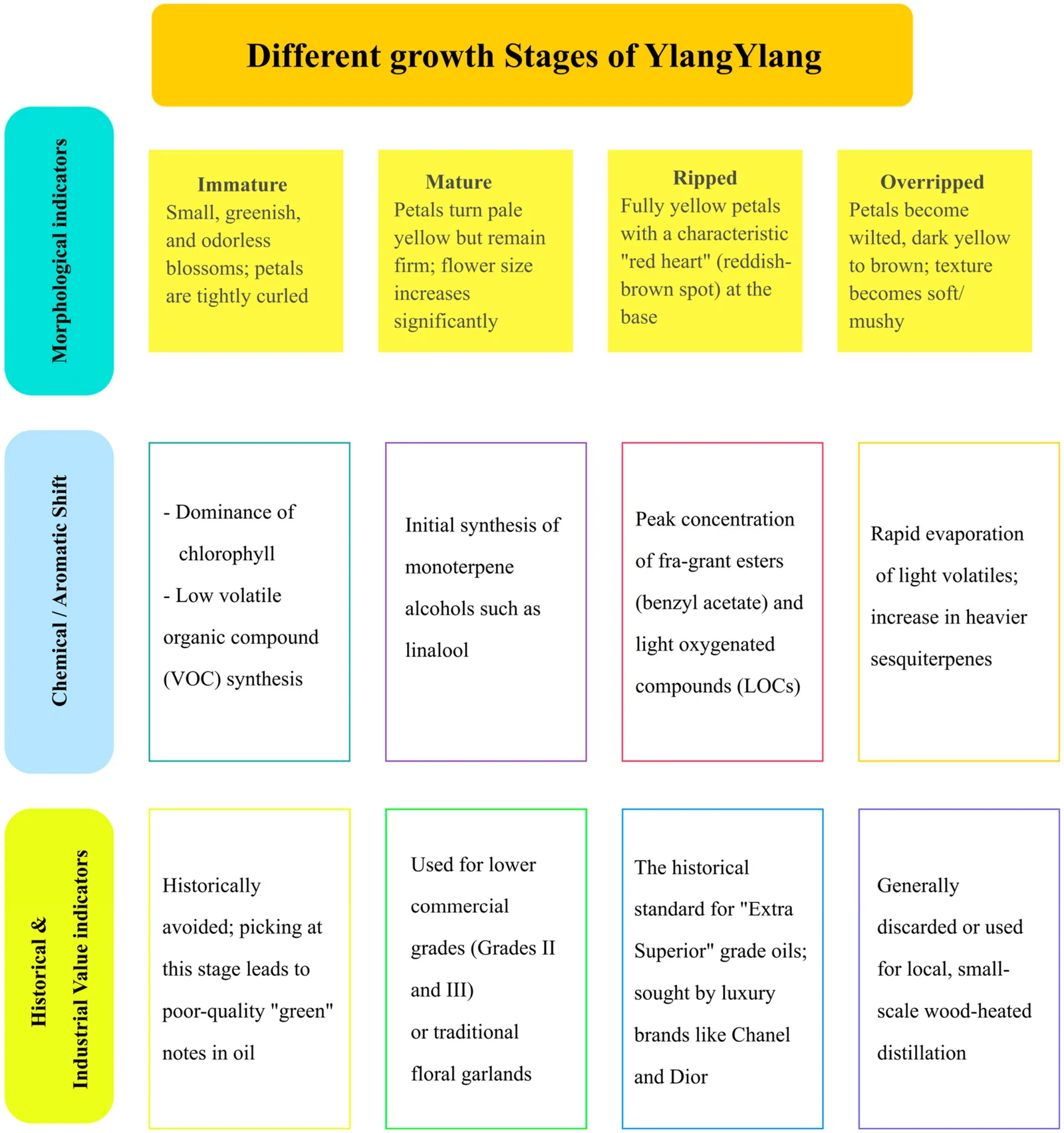
Different growth stages of the *C. odorata* tree.

These species mostly grow in Southeast Asia to heights between 50 and 65 feet (15–20 m). However, under ideal conditions in their native tropical Asia, their height has been recorded at nearly 30 m. Morphologically, *C. odorata* has alternate leaves, which are deep green and glossy with noticeably wavy edges, and ovate, oblong, long, drooping branches [1,4]. The *C. odorata* tree is primarily found in Java, where its flowers bloom year-round. YYEO is mainly produced in Indonesia, Madagascar, the Comoros Islands, and Mayotte, where *C. odorata* trees are cultivated for commercial purposes. Acidic soils of rainforest areas are preferred for *C. odorata* to grow efficiently. First, plants are raised from seeds, which are sown in nursery beds in June/July. This process usually takes place two months before the rainy season. Germination occurs in two months, when the plants’ height is about 50 cm. Then, the seedlings are transplanted to the field with a spacing of about 5–6 m, allowing the trees to grow in an umbrella shape. About five years later, the trees start to yield flowers for up to about 30 years. Different growth stages of *C. odorata* are briefly shown in Figure 2.

Flowering is low during December to March and July to September and is high during April to June and October to December [5]. A Philippine expression of “Along-Ilang”, which means “hanging” or “fluttering item”, was used to refer to the *C. odorata* trees because of the drooping nature of the tree’s flowers [6]. The flowers are highly fragrant and star-shaped. Each flower usually has slightly thick petals whose color changes as they mature from greenish yellow when young to deep yellow or yellow–brown at maturity, often with a reddish-purple center. They grow in axillary, umbrella-like clusters, with 4–12 flowers [1,7]. A brief integrated profile of *C. odorata* is shown in Table 1.

*C. odorata* is an essential plant resource and a key cash crop, especially in the Comoros Islands, which are currently the world’s top producers. In the Comoros, the harvesting of these aromatic flowers brings in roughly €1.5 million each year, representing 11% of the national income and offering employment to 10% of the working population, including hundreds of thousands of individuals engaged in production, harvesting, and distillation [8]. About 96% of the Comorian yield is directly exported to France, where it is a fundamental component of the luxury perfume market, used by renowned brands like Chanel, Dior, and Guerlain. Current global production is estimated at 50–100 metric tons per year, while the prices of essential oils range from 50 to 180 €/kg [9]. In addition to its major role in perfumery, YYEO is also used in the food industry as a flavoring agent and is classified as Generally Recognized as Safe (GRAS) for food use. Reported maximum use levels reach approximately 25 ppm in chewing gum, 2.9 ppm in baked goods and hard candies, 1.4 ppm in frozen dairy products, and 0.95 ppm in non-alcoholic beverages [3]. These quantitative data, together with its use across several food categories, demonstrate the established relevance of YYEO as a natural flavoring ingredient. A literature search was conducted using the Scopus database to provide an overview of the research landscape related to YYEO essential oil. The search was performed in the Title, Abstract, and Keywords fields using the following query:

TITLE-ABS-KEY(“ylang ylang”) AND PUBYEAR > 2009 AND PUBYEAR < 2027

**Figure 3:**
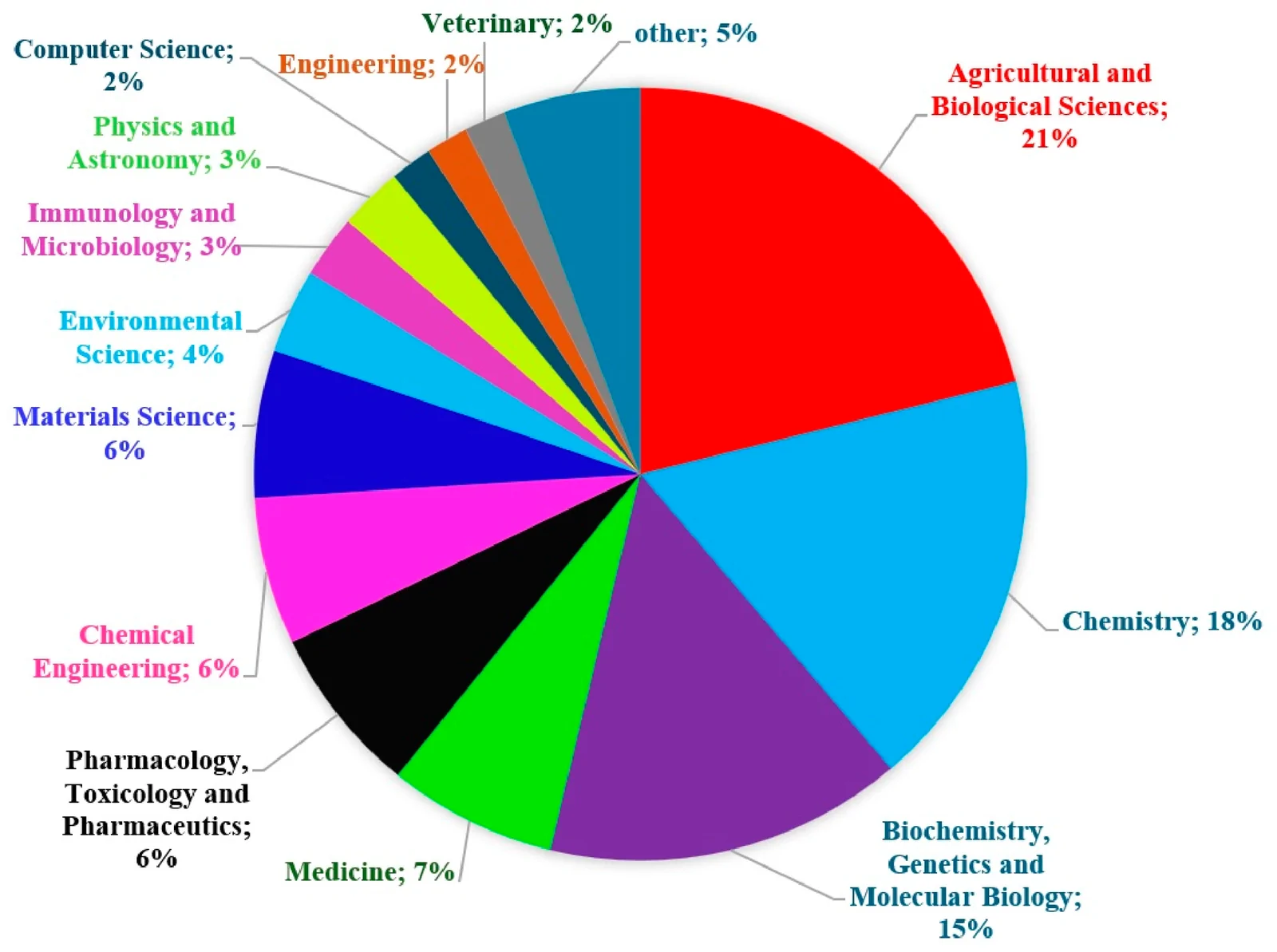
Different applications of YYEO.

The search included all document types and covered publications from 2010 to 2026. The literature search and the data used to generate Figure 3 were updated on August 20, 2026. According to the Scopus subject-area classification, all extracted results were analyzed. The number of publications assigned to each subject area was extracted, and the corresponding percentages were calculated based on the total number of publications. The processed data was subsequently used to generate the pie chart shown in Figure 3.

While several review articles have been published on YYEO, they are predominantly fragmented in their scope, often focusing on isolated aspects such as chemical profiling and pharmacological bioactivities [10] or overlooking YYEO chemical composition without paying attention to the remarkable effect of extraction methods on resulted essential oil quality [1]. This fragmented approach limits the holistic understanding required to correlate process parameters with the final properties and chemical composition of the essential oil. The existing literature fails to provide a systematic link between the operational conditions of green extraction techniques such as Supercritical Fluid Extraction (SFE) and the preservation of the volatile bioactive markers that directly affect the efficacy of essential oil. To bridge this critical knowledge gap, this review presents a comprehensive and integrated analysis of conventional and green extraction techniques, chemical composition, different properties, and applications. As a result, this work provides a cohesive resource that enables researchers to identify optimal processing conditions more efficiently and offers a deeper mechanistic insight into the relationship between extraction design and final product quality.

## 2. Essential Oil Extraction

### 2.1. Conventional Distillation Methods

#### 2.1.1. Hydrodistillation

Hydrodistillation (HD) is a key physical technique used to extract volatile secondary metabolites from the fresh, mature flowers of *C. odorata* [11]. In typical laboratory environments, this method typically uses a Clevenger-type apparatus, whereas in industrial settings, large alembic stills are preferred. The process involves combining the botanical material, which is often kept at around 53% moisture content, with water in an extraction vessel. This mixture is then heated, traditionally using wood (WHD) or more contemporary electric heating elements, to achieve a boiling temperature of 100 °C [8]. The high temperature causes the essential oil (EO) to vaporize and escape from the solid plant matrix through azeotropic distillation [12]. The mixture is then heated, either by traditional wood methods or by modern electric heating elements, until it reaches 100 °C. This high temperature causes the vaporization of the essential oil (EO), allowing it to escape from the solid plant material via azeotropic distillation. The blend of steam and aromatic vapors passes through a distillation head into a condenser, where a cooling system, such as a stainless-steel coil or a swan neck, condenses the vapors into liquid. This liquid is subsequently collected in an essence, or oil–water separator, where the oil and water naturally separate into two distinct layers due to their differing densities and immiscibility, with YYEO usually forming the upper layer.

Though hydrodistillation is widely recognized as a commercial standard worldwide, it is viewed as a time- and energy-intensive process, typically taking about 19 h for a complete extraction cycle [13]. Moreover, this method employs a sequential fractionation approach, in which the oil is collected at various stages to produce different grades of the essential oil [8,14]. These grades are categorized by density and chemical characteristics; early fractions are highly valued in luxury perfumery for their high concentration of light, oxygenated compounds such as linalool, p-cresyl methyl ether, and benzyl acetate, while later fractions are largely composed of heavier, less volatile sesquiterpene hydrocarbons such as germacrene D [8,15,16,17]. In addition, other factors such as acid and ester value, refractive index, and optical rotation can be further determining parameters [12].

Although hydrodistillation is a common method for essential oil extraction, there are several key environmental challenges associated with the traditional HD process. Prolonged exposure to elevated temperatures and water in traditional hot-water extraction commonly results in the thermal and hydrolytic degradation of delicate components, especially esters, ultimately leading to considerably lower benzyl acetate yields than with more advanced extraction techniques [18,19,20].

In addition to the degradation of the elements, environmental issues are another important drawback. For instance, as steam generation and extraction are highly time-consuming, the HD process will be energy-intensive. The demand for energy to power the traditional distillation units may be met by exploiting forest biomass. Moreover, insufficient amounts of oil yield mean that there is a greater need for planted areas or acreage to produce the same amounts of essential oil, which indirectly contributes to land-use pressure and deforestation [21]. Reliance on traditional energy sources will result in higher amounts of CO_2_ emissions compared to other modern technologies, such as microwave-assisted hydrodistillation [22]. In addition, this method requires a steady supply of cool water for either the extraction of solvent or cooling, leading to ecotoxicity and aquatic pollution of local rivers through wastes containing secondary metabolites and organic compounds released [21,23]. To address these imperative environmental issues, it is important to transition toward green distillation technologies that prioritize efficient energy consumption, reduced waste, and ecological sustainability.

**Figure 4:**
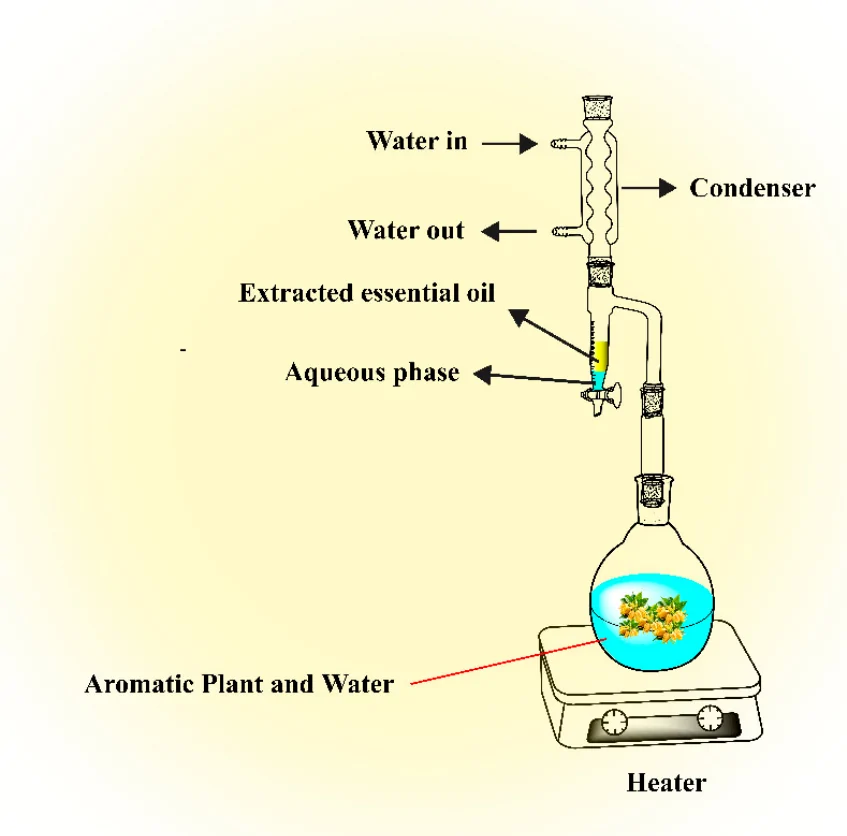
Hydrodistillation method for essential oil extraction.

The hydrodistillation method may be developed by adding an extra step, which is adding absorbents to capture some specific compounds [24]. Solid additives such as activated carbon can be used to bind specific molecules in the vapor phase to make the extracted oil more purified and neater, with an aromatic profile [25,26]. Although adding this step can be useful in terms of lower processing temperature [27], versatility [28], and selectivity [24,25], adsorption materials may be harmful to the environment if they are not selected and regenerated properly [29]. Figure 4 shows the basic hydrodistillation method to extract essential oils.

#### 2.1.2. Steam–Water Distillation

**Figure 5:**
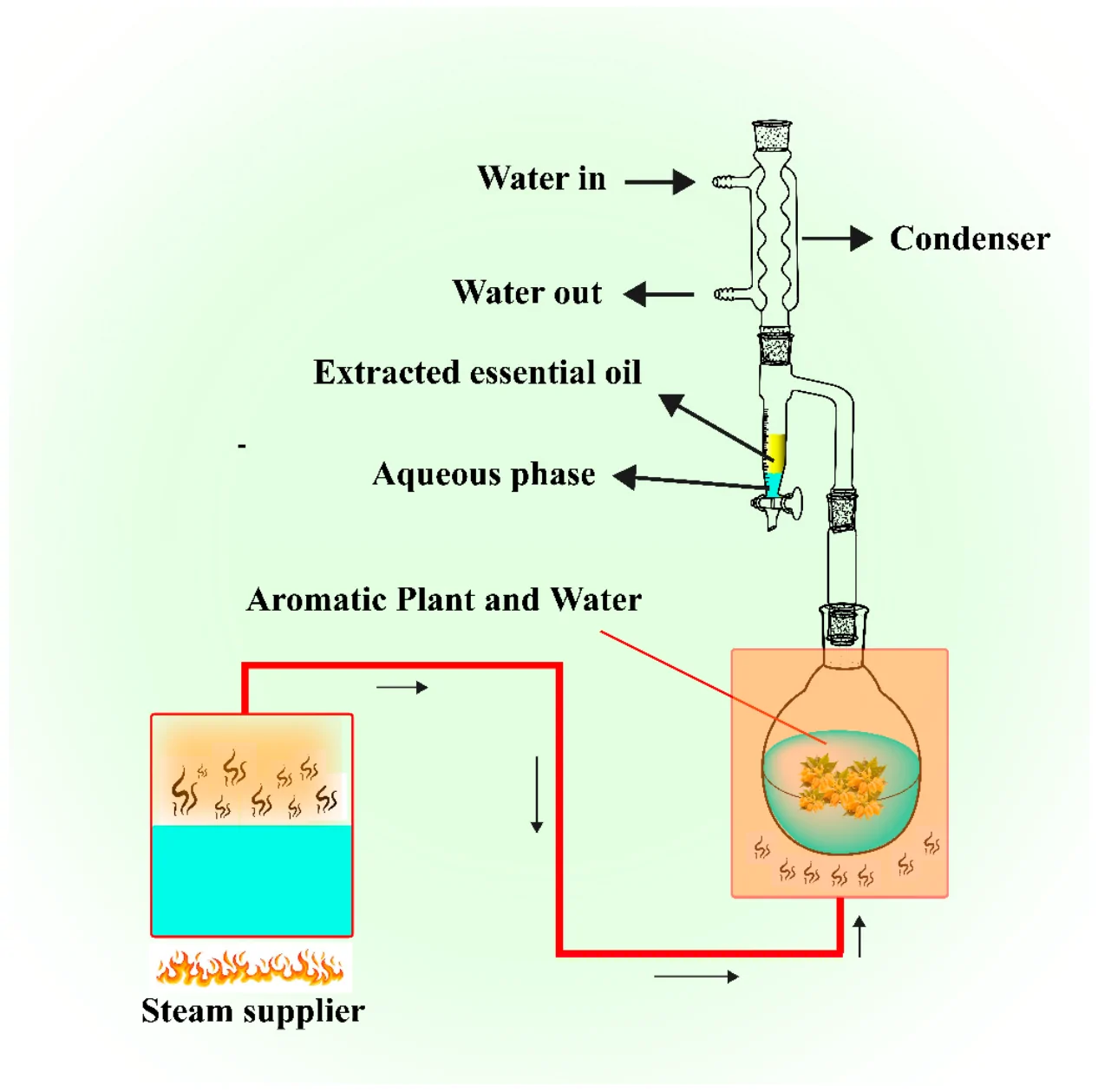
Schematic of steam distillation for essential oil extraction.

Steam distillation is a standard traditional industrial method for the extraction of essential oils with high quality, especially for plants such as tea tree, lemon, and rosemary, which have high amounts of essential oil [30]. As shown in Figure 5, the isolation of essential oils is done by passing steam through botanical matrices, including seeds, leaves, and blossoms, to vaporize volatile fractions, followed by condensation and phase separation, which is based on the density of the portions. After condensation, oils float on the water, leading to easy separation of oils [31,32]. Although this method is valuable for producing oils with high purity, which is suitable for the cosmetic and food industry, it is essential to either regulate the temperature to prevent degradation of sensitive constituents because of elevated temperatures or choose an alternative method for extraction [33,34]. Moreover, high energy consumption for high temperatures can be a disadvantage for energy-intensive operations [35]. Steam distillation can handle large-scale operations effectively, making it a proper method for commercial use. However, initial high investments to prepare industrial-scale condensers, boilers, and separators are needed, which is one of the disadvantages of this technique [36,37]. In addition, high resource footprints of water and energy present significant operational hurdles. Recent literature has focused on optimizing strategies to conserve resources and integrate renewable energies to improve the ecological profile of the processes [38,39].

#### 2.1.3. Solvent Extraction

**Figure 6:**
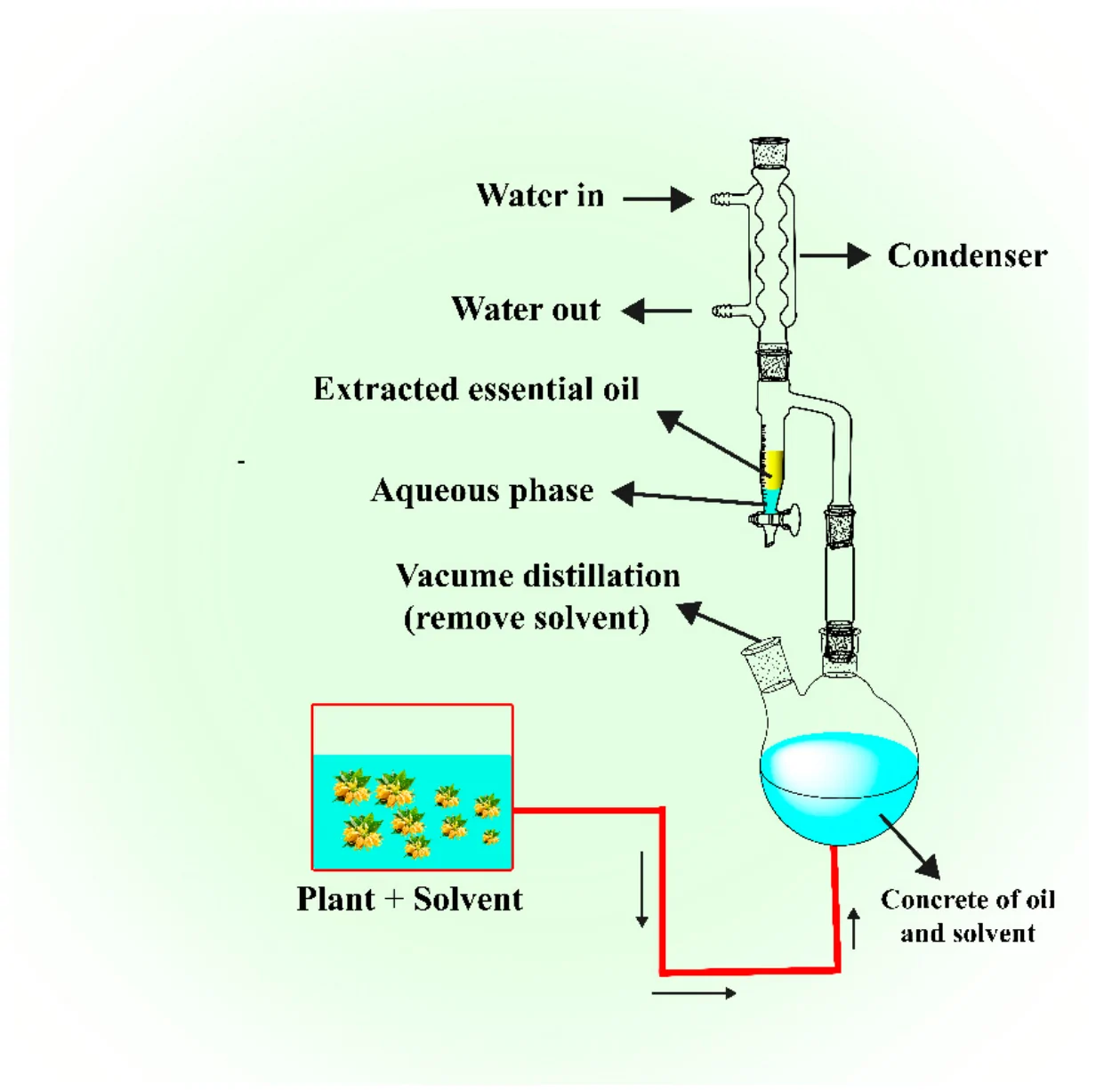
Solvent extraction of essential oil.

Solvent extraction is a versatile method to extract essential oils from plants that have a small proportion of oil content, such as jasmine flowers and roses [40]. Several solvents such as hexane, ethanol, and supercritical carbon dioxide have been used to dissolve the plant’s oil, which is then evaporated to obtain pure oil [41,42]. Several advantages are reported for the solvent extraction method. The most important advantage of this method is the potential to extract oil from the flowers that cannot tolerate high temperatures. Moreover, complex aromatic structures that have a series of fragrances can be extracted. The quality of oil extracted by this method is much higher than that of hydrodistillation. However, when it comes to the food industry and cosmetics, the potential solvent residues pose some challenges to this method [43,44]. Solvent extraction can be done on an industrial scale as it is efficient and scalable, and large amounts of materials can be processed, making it a cost-effective method of extraction [45,46]. However, large amounts of organic solvents, which are used to dissolve oils from plants, can have environmental impacts and higher costs. Therefore, procedures should be managed to recover solvents and any kind of disposal to prevent environmental impacts [47]. Figure 6 illustrates the basic process of solvent extraction.

### 2.2. Innovative Green Technologies:

#### 2.2.1. Microwave-Assisted Distillation (MAD)

**Figure 7:**
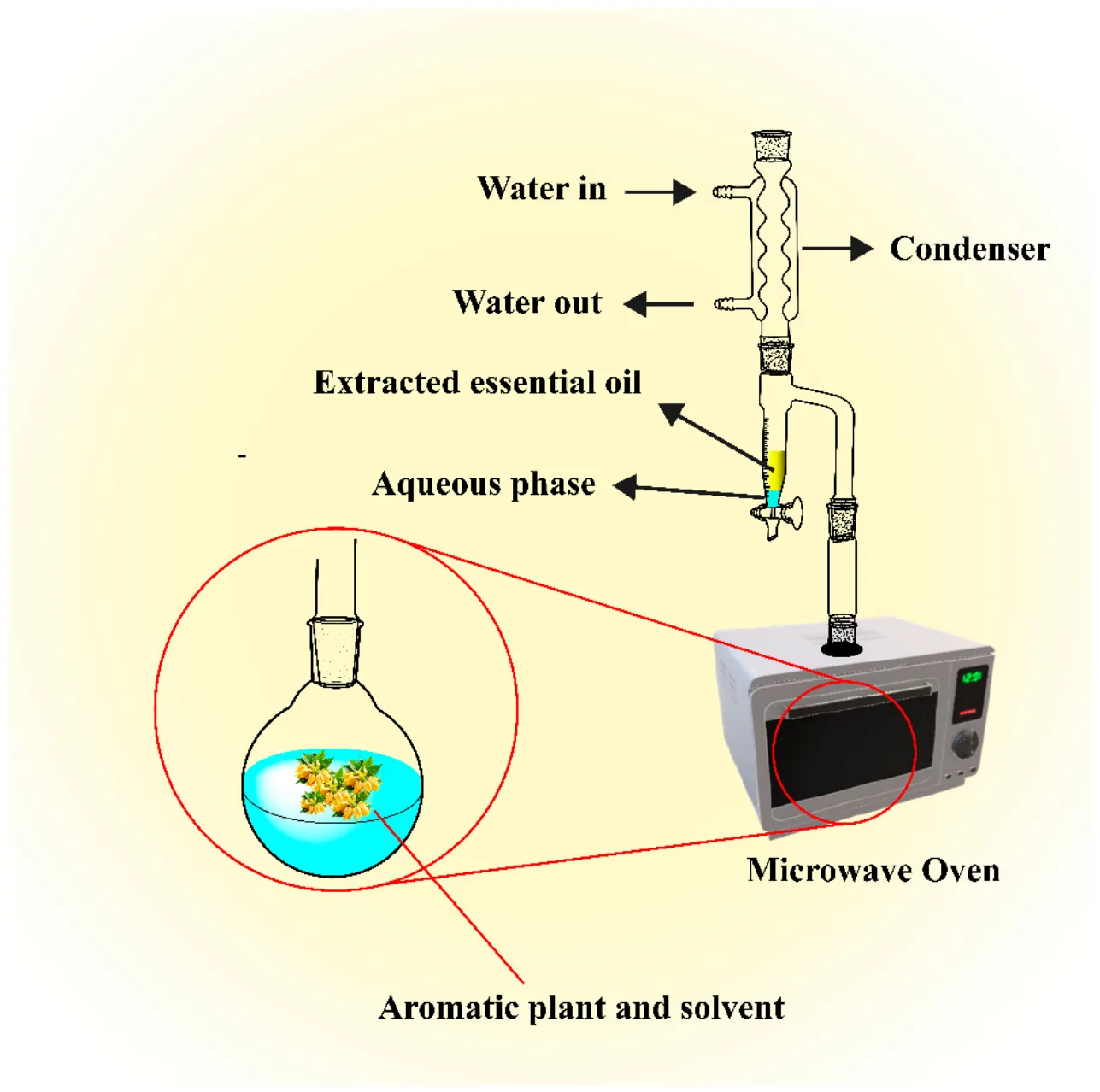
MAD process of essential oil extraction.

Microwave-Assisted Distillation (MAD) is an efficient, modern, eco-friendly method for essential oil extraction. This process utilizes a unique heating mechanism that targets polar molecules, such as the moisture that is naturally in plant materials. When microwave heating starts, polar molecules begin to vibrate, and their temperature rises sharply, as well as the pressure inside the plant cells. This internal pressure eventually promotes the oil glands and receptacles to expand and rupture, finally leading to the release of volatile secondary metabolites that readily evaporate via azeotropic distillation [12,14,48,49]. Typically, as shown in Figure 7, experimental setups use a microwave oven operating at power levels ranging from 250 W to 1000 W, paired with a cooling system to condense the steam and aromatic vapors produced [50,51].

Using the MAD method will help to combine efficiency with sustainability [48]. Several research demonstrated the effectiveness of this method, which is one of the most appropriate means of distillation of essential oils. The results showed that the remarkable decrease in processing time is the main benefit of MAD compared to traditional methods such as wood-heated distillation (WHD) or laboratory-scale distillation (LD) [19,49]. For instance, research demonstrated that YYEO can be fully extracted in just 40 min using MAD, whereas conventional hydrodistillation typically takes 19 to 20 h [50]. In addition, MAD demonstrated significantly higher efficiency, providing an extraction yield that is 29% greater than WHD and 23% more than LD, and some others showed more than 40% essential oil yield compared to traditional methods [8,52]. Apart from its efficiency, enhancement in the quality of the obtained essential oil is also reported, highlighting a higher proportion of linalool and benzyl acetate as odoriferous light oxygenated compounds (LOCs), which are essential for the distinctive YYEO aroma [1]. Analytical results reveal that LOCs account for about 81.23% of the SFME extract, compared with much lower percentages in hydrodistillation (69.94%) and steam distillation (57.98%) [12]. As microwaves can target molecules with higher dipole moments, such as alcohols and aromatic esters, their integrity will be retained, and they will experience less thermal and hydrolytic degradation [51]. This ability to select specific oxygenated molecules may come from strong interactions between microwaves and oxygenated compounds, whose C-O bonds are intensely polarized. This phenomenon is shown for other similar compounds [53,54]. Although this method has several advantages, some limitations, such as microwave power, the ratio of solvent to raw materials, and extraction time, can influence the efficiency of this process [55]. In addition, the need for specialized apparatus and the possibility of uneven heating are other challenges in industrial applications [51]. Table 2 reviews some literatures utilized MAE to extract different essential oils. The reported studies demonstrate that microwave-assisted techniques can substantially reduce extraction time and may provide favorable yields under optimized conditions. However, direct comparison of yield values across studies should be interpreted cautiously because the plant species, material basis, extraction conditions, and yield definitions differ. Although this review primarily focuses on ylang-ylang essential oil, the number of studies specifically investigating microwave-assisted extraction of YYEO remains limited. Therefore, selected studies involving other aromatic plants and essential oils are included in Table 2 solely to provide methodological context regarding the operating conditions and potential applications of microwave-assisted extraction. These data should not be directly compared with YYEO results or interpreted as evidence of the extraction performance of YYEO.

Commercially, MAD represents a significant transformation in production grading: whereas traditional methods typically produce around 70% low-value “Grade III” fractions, MAD achieves 81% in high-value “Extra Superior” and “Extra” grades. From an environmental standpoint, MAD is viewed as a “green” technology that substantially lessens ecological impact. In areas such as the Comoro Islands, implementing MAD could help alleviate significant deforestation by eliminating the need for large amounts of wood (about 1 ton per distillation) to heat conventional stills, while also cutting water use for cooling by 10–20 m^3^ per cycle. Although the initial costs of durable microwave generators and specialized reactors are steep, continuous technological progress and successful pilot-scale projects indicate a feasible route to wider industrial integration in high-end perfumery, aromatherapy, and contemporary materials science.

#### 2.2.2. Supercritical Fluid Extraction (SFE)

**Figure 8:**
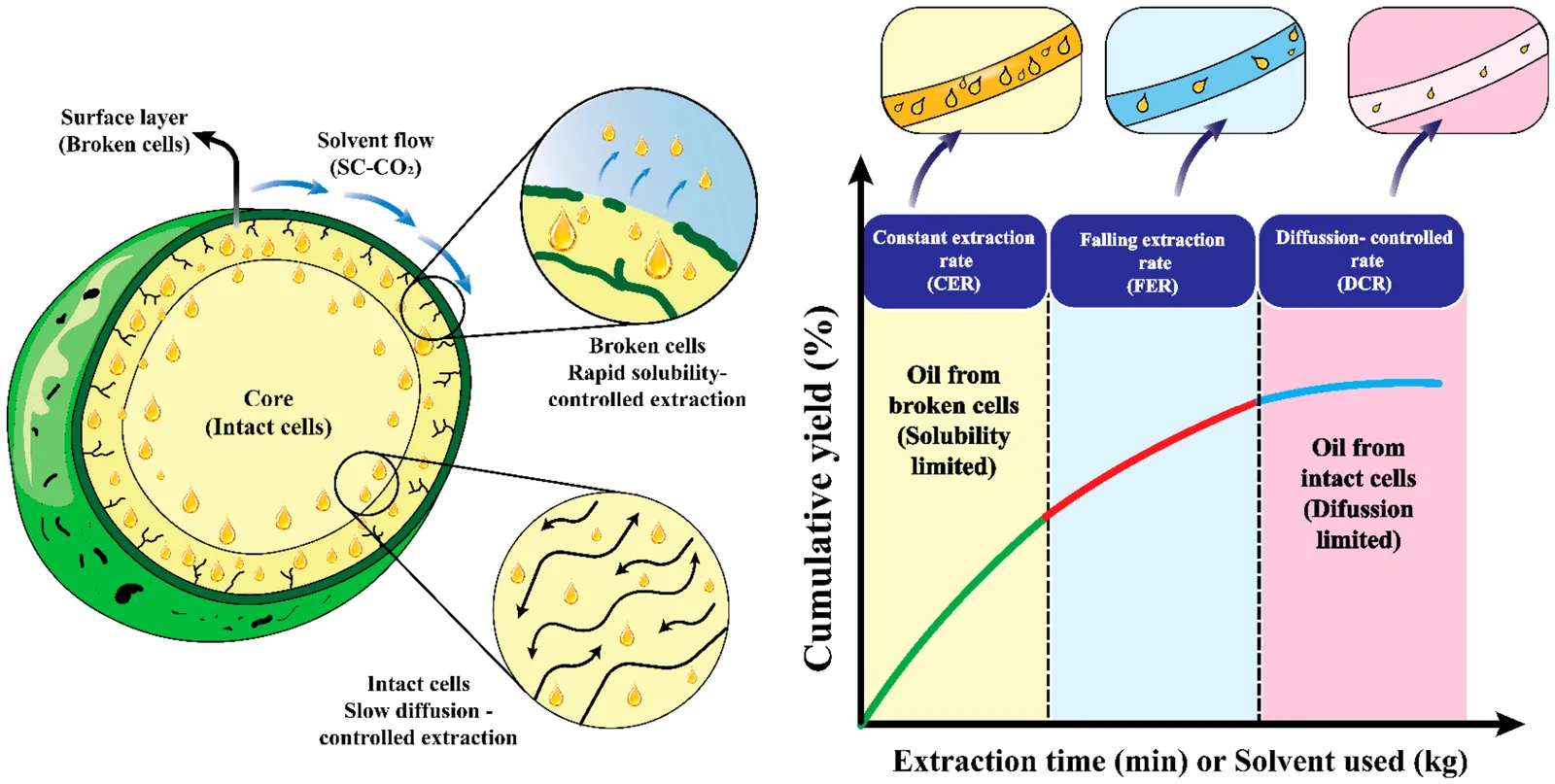
The Broken and Intact Cell (BIC) model for supercritical fluid extraction.

Supercritical fluid extraction (SFE) is a sophisticated, advanced approach to authentically extracting essential oils, as it can reduce the process time, consume less amount of organic solvent, produce a cleaner extract, and is environmentally friendly [65]. Among several supercritical fluids, supercritical CO_2_ is widely used for extraction as it is easy to handle, nontoxic, easily available in bulk quantities with high purity, and non-flammable [66,67,68]. Huang et al. [69] suggested a theoretical framework for the mechanism of SFE. Based on their theory, the mechanism of SFE consists of a multi-stage mass transfer process that occurs when supercritical CO_2_ flows in a fixed bed of porous plant material. Therefore, an external fluid film will be formed when solvent diffuses into the matrix pores and is adsorbed onto the particle surface. Solvent–solute interactions lead to the dissolution of target analyte molecules that are then convectively transported through the pores. Transportation of the dissolved solute into the bulk supercritical fluid phase occurs while diffusion of dissolved solute out of the internal matrix occurs. Finally, the solute precipitates for collection as the fluid exits the extraction unit and is depressurized to a gaseous state. These complex kinetics of SFE are described by the Broken and Intact Cell (BIC) approach, shown in Figure 8, separating extraction into three distinct periods: an initial constant extraction rate (CER) dominated by easily accessible surface oil, a falling extraction rate (FER) transition, and a final diffusion-controlled (DC) period where less accessible solute is slowly recovered from intact internal cells.

SFE can preserve thermally sensitive and intricate “top note” volatile compounds, which are usually degraded or lost in conventional methods. Moreover, SFE is known as a sustainable method due to the use of CO_2,_ which is an environmentally friendly, non-toxic material, having no harmful residues in final products [70,71]. The application of SFE specifically to YYEO remains relatively limited. Therefore, to provide methodological context and illustrate the range of operating conditions reported for essential-oil extraction, Table 3 also includes selected studies involving other aromatic plant materials. These studies are presented as methodological examples and should not be interpreted as direct evidence of YYEO extraction performance or used for quantitative comparison with YYEO.

**Figure 9:**
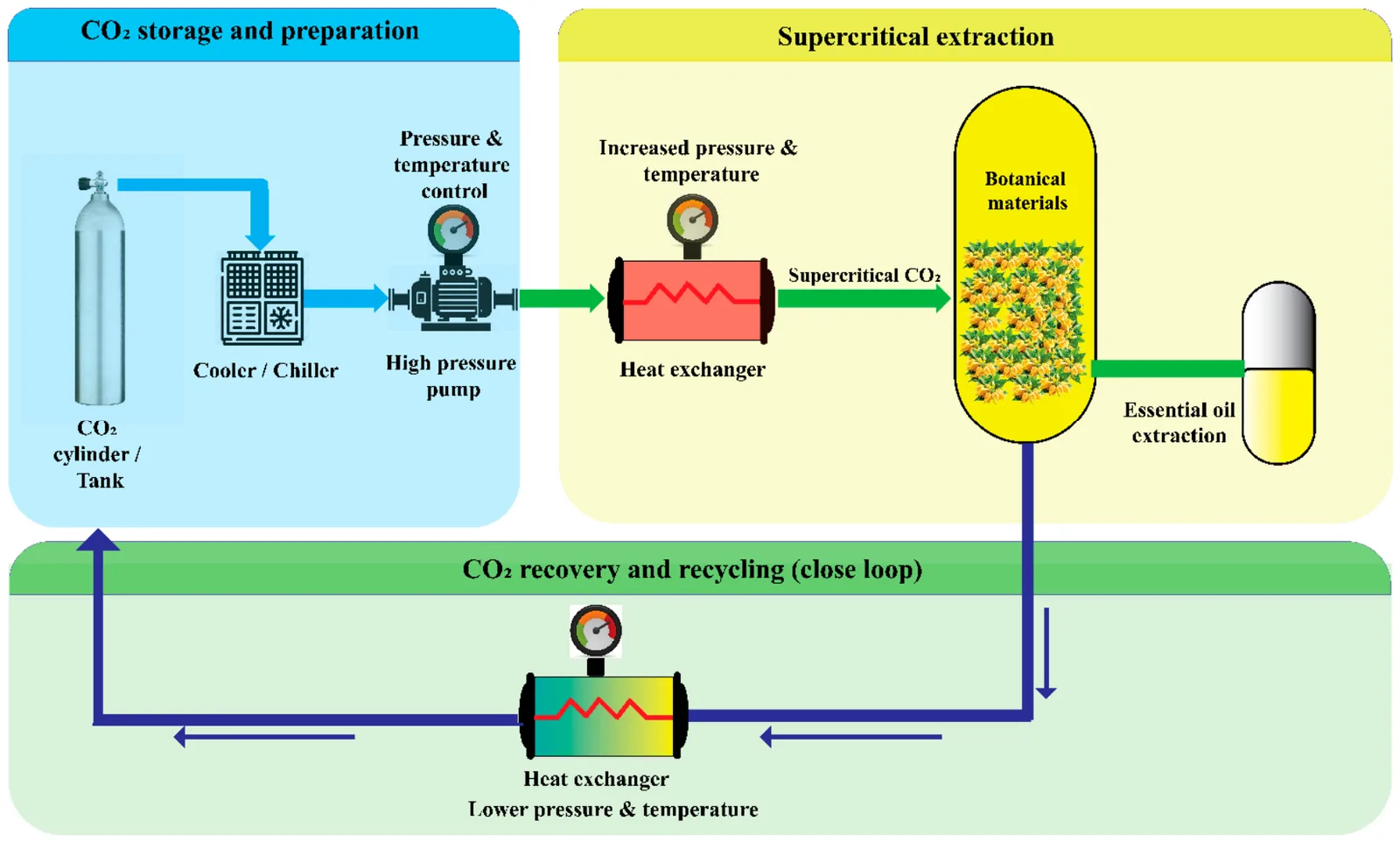
SFE procedure of essential oil extraction.

This method strikes a balance between efficiency, extraction quality, and environmental considerations that highlight the potential of this process [51,84]. However, pressure and temperature should be controlled precisely, adding to the complexity of the procedure [85]. Furthermore, there are challenges with maintaining SFE systems in less industrialized and developed settings, limiting their development worldwide [86]. Figure 9 shows the SFE procedure to extract essential oils.

#### 2.2.3. Ultrasound-Assisted Extraction (UAE)

**Figure 10:**
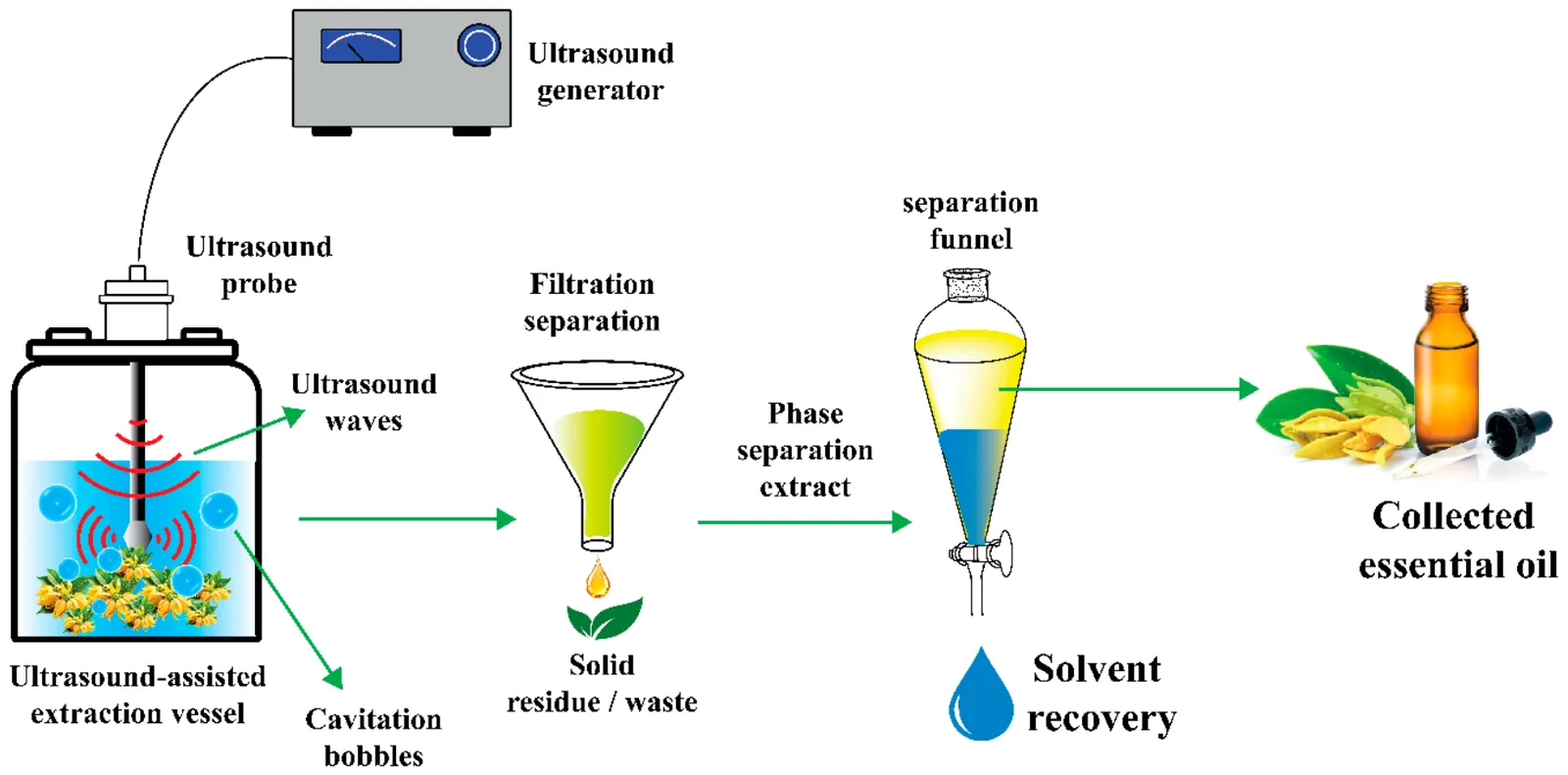
UAE method for essential oil extraction.

The transition toward “green” analytical chemistry has catalyzed the development of innovative extraction technologies. Among innovative green extraction technologies, ultrasound-assisted extraction (UAE) has emerged as a promising technique for the isolation of essential oils (EOs) from various botanical matrices, aimed at reducing environmental toxicity while maintaining high industrial throughput and a sustainable alternative to traditional methods [87,88]. In this method, ultrasound waves, typically ranging from 20 kHz to 100 kHz, determine the efficacy of the extraction by creating a physical phenomenon of acoustic cavitation. When ultrasonic waves spread through the solvent, they generate alternating cycles of high and low pressure [89]. During the low-pressure cycle, minute vacuum bubbles or cavities are formed, which then collapse intensely during the high-pressure cycle. When implosion occurs, extreme localized microjets and shock waves will be created, leading to significant shear forces [90,91]. Cavitation can facilitate processes through its mechanical impacts. For instance, sonoporation, which can increase the permeability of cell membranes, expansion and contraction of plant tissues, called the “sponge effect”, and total cell wall disruption [92,93]. These mechanisms can collectively increase the rate of mass transfer of volatile components from the plant’s internal glands into the surrounding solvent and enhance the mass transfer of volatile secondary metabolites from the internal glandular structures of the plant into the surrounding solvent [89]. The UAE can reduce the process time significantly from hours to just mere minutes, while keeping the operation temperature at low levels, contributing to the chemical integrity of thermosensitive components, such as monoterpenes and oxygenated sesquiterpenes [94,95]. Furthermore, the integration of the UAE may be helpful for waste reduction and energy consumption. UAE utilizes “green” solvents, such as Natural Deep Eutectic Solvents (NADES), which have synergistic effects on higher yields of high purity [87,96]. However, the optimization of UAE is a multifaceted challenge, as efficiency is governed by several independent variables, including ultrasonic power, frequency, temperature, and the solid-to-liquid ratio [97,98,99]. Moreover, it is vital to have control over operating conditions, as uneven cavitation can have negative impacts on consistent extraction [100]. Figure 10 shows the UAE method to extract essential oils.

#### 2.2.4. Summary of Extraction Technologies

The extraction of essential oils has evolved from traditional hydrodistillation (HD), which is characterized by long time of the process and potential thermal degradation of delicate esters, toward “Green” technology of extraction such as Microwave-Assisted Distillation (MAD), Ultrasound-Assisted (UAE), and Supercritical Fluid Extraction (SFE), offering high-value “Extra” and “Superior” phytochemical grades while significant reductions in energy consumption and extraction time. Specifically, MAD utilizes selective heating to rupture oil glands rapidly, while SFE provides a solvent-free path to high-purity and high-quality extracts. These advancements not only enhance the chemical integrity of the important constituents, such as linalool and germacrene-D, but also align essential oils production with global sustainability goals by minimizing the carbon and water footprints of the process. Comparing the available findings, it becomes evident that no single extraction technology can be regarded as universally optimal for YYEO production. Conventional hydrodistillation remains relevant because of its technological simplicity, established industrial use, and its ability to obtain sequential fractions corresponding to the commercial grades of YYEO. However, long processing time, high demand for heat and cooling water, and probable thermal or hydrolytic degradation of sensitive volatile compounds may be regarded as its significant limitations, particularly when the preservation of light oxygenated constituents is a priority. It is evident that replacement of green extraction technologies with conventional distillation methods is not simple, but they can be process-specific alternatives whose suitability depends on intended product quality, production scale, and economic constraints.

Table 4 highlights a qualitative comparative analysis of traditional and Innovative extraction methods for the extraction of different essential oils.

## 3. Phytochemical Profile and Structural Analysis

The chemical composition of YYEO is complex, and different parameters such as geographical origin, maturity of the flowers, extraction techniques and time, and harvesting conditions can affect the quality of the extracted oil [101,102,103]. Therefore, the relative abundance of individual constituents reported in the literature should not be interpreted as fixed values. Across more than 100 constituents reported in *C. odorata* essential oils and extracts, some individual analytical profiles smaller number of volatile constituents.

The volatile fraction of YYEO is usually dominated by monoterpenes and sesquiterpenes, together with oxygenated compounds, including alcohols and esters. The most important oxygenated compound has been reported to be Linalool and may account for approximately 28%, as well as aromatic esters such as benzyl acetate, methyl benzoate, and geranyl acetate. On the other hand, up to 63% of the essential oil comprises a hydrocarbon fraction mainly containing sesquiterpenes, in particular germacrene D and β-caryophyllene. These sesquiterpenes, which are mostly heavier, along with other sesquiterpenes, such as α-humulene and farnesene, have been mainly used in textile applications as they are less volatile than monoterpenes, making them a natural fixative that causes a lower rate of release of essential oil and efficient repellency properties [104].

**Figure 11:**
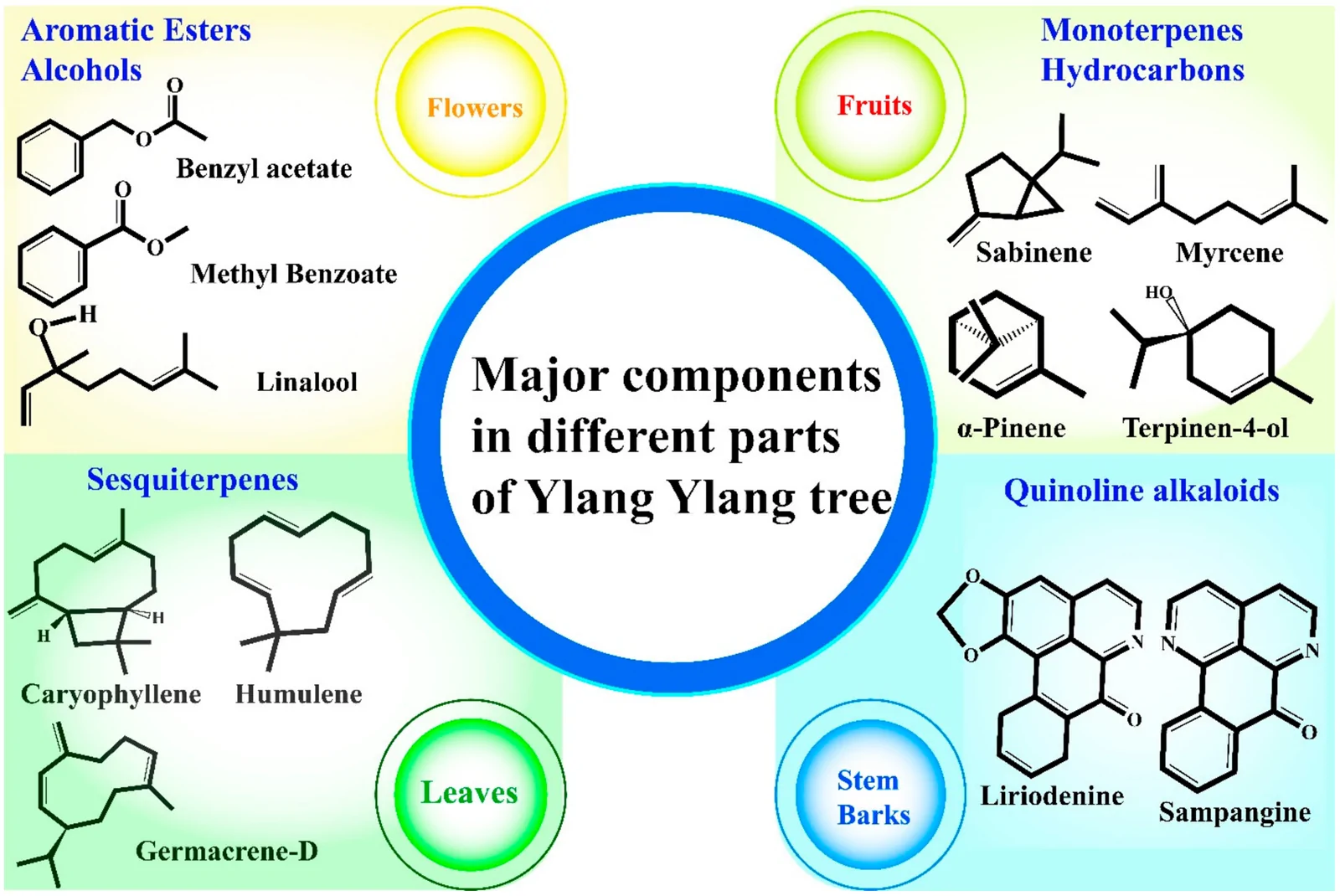
Main components extracted from different parts of the *C. odorata* tree.

To date, there are five categories with respective quality and application for YYEO base factors mentioned above, including extra, first, second, third, and complete. Up to 40% of the distillate, which is collected in the first hours of distillation, is the extra and first grades [105]. These grades of YYEO contain a large amount of volatile, strong odoriferous compounds such as linalool, p-cresyl methyl ether, and benzyl acetate, making greater odor. This property makes the extra grade a promising option in the fragrance industry [1,6]. In terms of other grades, they comprise smaller amounts of volatile compounds of sesquiterpenes, namely caryophyllene, germacrene D, and α-farnesene, that are used to produce soaps and cosmetics [105]. Complete grades contain either one of the grades or all of them together, and their usage depends on the manufacturer’s decision [106]. There is also a large array of non-volatile compounds having strong bioactivity. These bioactive secondary metabolites, such as alkaloids, flavonoids, phenols, tannins, and glycosides, are mainly identified through solvent extraction of the plant’s bark, seeds, and fruits [1]. These compounds usually have diverse bioactivities such as antimicrobial, anti-inflammatory, anti-cancer, antibiofilm, anti-diabetic, and anti-vector [107]. Some important components of YYEO extracted from different parts of the *C. odorata* tree are shown in Figure 11.

## 4. Different Applications of YYEO

### 4.1. Insecticide Properties of YYEO Against Vector-Borne Diseases

The insecticidal and repellent potential of YYEO signifies a transformative frontier in sustainable pest management, bridging the gap between traditional ethnomedicine and modern biopesticide engineering. As discussed, the phytochemical landscape of YYEO is highly variable, and more than 100 compounds have been reported in different studies. However, individual analytical profiles typically identify a smaller number of major volatile constituents, primarily belonging to monoterpenes, sesquiterpenes, and phenylpropanoids. These complex compounds are the main drivers of YYEO’s biological properties. The insecticidal activity of the YYEO is rooted in several volatile constituents, including linalool, benzyl acetate, and geraniol, although the overall activity may result from the combined or synergetic effects of multiple compounds [1,15,108]. Although flower oil contains aromatic esters, leaf essential oil is mainly dominated by caryophyllene (30.3%) and humulene (13.4%), making it a promising option for post-harvest pests [109]. Therefore, preserving special compounds of YYEO can be useful when it comes to repellency properties. In this regard, advanced green extraction, such as MAD, UAE, and SFE, can preserve more than 81% of the LOCs, compared with 69.94 and 57.98% obtained by hydrodistillation and steam distillation, respectively. These oxygenated volatile constituents may affect biological activity and characteristic aroma, resulting in the final performance of the oil. However, insecticide properties and repellency should be experimentally determined rather than inferred solely from chemical composition [8,12,110].

Insect repellents generally decrease host attraction or interfere with the behavioral and sensory processes used by insects to locate and approach a host. These agents, which usually have a synthetic or botanical origin, may be in contrast with some insecticides that employ neurotoxic mechanisms to impact the insect’s nervous system, leading to mortality. Some pyrethroids can show both repellent and insecticide effects. Natural pyrethrins are derived from *Chrysanthemum* species, whereas compounds such as permethrin are synthetic pyrethroids [111]. Vector-borne diseases such as dengue, malaria, and Zika have put public health in danger in the past and present. As they cannot communicate with people directly, they will prevail when proper conditions, such as the environment and hosts, are provided [112]. Dengue virus (DENV), which is found in tropical and subtropical climates worldwide, can cause either mild illness or, in some cases, more severe illnesses and even death. Based on research, more than half of the world population is in danger of DENV, with an estimated 390 million infections occurring annually, of which 96 million manifest clinically [113]. Zika virus (ZIKAV) is another dangerous virus that is mainly transmitted by *Aedes* mosquito bites, causing infection and microcephaly in infants (smaller than normal head size) during pregnancy, congenital malformations, the neurological disorder Guillain–Barré syndrome (GBS), as well as preterm birth and miscarriage [114,115]. A systematic review demonstrated that the prevalence of microcephaly among infants whose mothers were infected with ZIKAV put them in danger of neurological disease, such as non-language cognitive delay [116]. According to the WHO’s latest World Malaria Report, 241 million malaria cases and 627,000 malaria deaths worldwide were reported in 2020, and there were 597,000 global malaria deaths at 13.7 deaths per 100,000 individuals in 2023. Worryingly, as there are no certain treatments and investments for these kinds of diseases yet, control over transmission can help to lower the fatality rate, especially in urban and semi-urban areas, which are far from instant medical care [117,118]. Historically, market domination of synthetic agents started with N, N-Diethyl-3-methylbenzamide (DEET) when the U.S. Army used it during World War II for its soldiers against insects in 1946. Following its civilian introduction in 1957, DEET has gained global publicity, with an estimated annual user base of 200 million people, including significant populations in the United States and the United Kingdom. It is commercially available in diverse formulations, ranging from aerosols and lotions to saturated wipes with concentrations spanning 5% to 100%. However, although it is an efficient repellent, its potential adverse effects on human health have prompted scientists to investigate proper, safer bio-based alternatives [111,119,120,121,122]. Therefore, there is growing interest in developing bio-based insecticides and repellent agents with favorable safety profiles and efficiency. However, when it comes to human health and environmental issues, their toxicity and environmental impacts should be checked individually. Traditionally, many plant-based insecticides have been used and developed, and several of them are under consideration to be more industrialized [121]. In a study, YYEO was encapsulated with urea formaldehyde in microcapsules having sizes of around 50–100 μm. Cotton fabric was treated with microcapsules containing 10 mL and 2.5 mL of YYEO. Then, treated and untreated cotton fabrics were assessed in terms of mosquito repellency against female adult *Aedes aegypti* mosquitoes, using the World Health Organization (WHO) cone test in five replicates. Results showed that the treated cotton fabric demonstrated repellency of more than 80% [123]. These green insecticides may be applied to textiles to give them repellent properties. However, while applying insecticides, it is important to pay attention to several characteristics mentioned in Table 5 [124,125].

The biological spectrum of YYEO’s activity is remarkably extensive, targeting major agricultural and medical vectors. In the realm of public health, essential oils from *C. odorata* flowers have proved remarkable larvicidal efficiency against *Aedes aegypti*, which is the main vector for *Dengue*, *Zika*, and *Culex* species [126,127]. Experimental bioassays on 82 commercial essential oils confirm that YYEO can reduce more than 95% of the peach fruit flies if it is used in a sustainable formulation [128]. Purista et al. [126] evaluated the effectiveness of ethanol extracts from *Zodia* leaves (Evodia suaveolens) and *C. odorata* flowers against Stage III–IV *Aedes aegypti* larvae. Ingredients were processed by the maceration method, and extracts were prepared at concentrations of 2.5% and 5%. Larval mortality was monitored at 6 h intervals over 24 h. After 24 h, all samples showed 100% larval mortality. Ethanol extracts of *Zodia* leaves and *C. odorata* flowers at concentrations as low as 2.5% are as effective as Temephos in eliminating *Aedes aegypti* larvae, suggesting their potential as viable botanical insecticides. Another study indicates that YYEO has high toxicity against C. quinquefasciatus larvae, with LC50 < 70 ppm. Rich in linalool and alpha-thujene, these readily available fragrance oils represent promising, sustainable ingredients for botanical formulations targeting public health pests [9]. Collectively, these findings highlight YYEO as a paramount candidate for the next generation of non-toxic, eco-friendly, high-performance botanical insecticides.

### 4.2. Psychological and Mood-Related Effects of YYEO

Essential oils’ effects on our brain can be explained by looking at the interaction between the brain and the EOs’ chemical structure and the signals that EOs send to the cells. Although this evaluation can provide a proper framework, empirical support is a must. From a neurobiological view, these effects are primarily linked to the interaction of odorant molecules with the olfactory system and its direct connections to brain regions involved in emotion and memory processing. Upon inhalation, volatile compounds bind to olfactory receptors in the nasal epithelium, initiating neural signals that are transmitted to the olfactory bulb and further along the olfactory tract. Unlike other sensory modalities, olfactory processing does not rely on an obligatory thalamic relay, but instead projects directly to limbic structures [1,129]. Sensory inputs are quickly evaluated by the amygdala, especially in relation to affective salience, whereas memory formation and associative learning are done by involvement of the hippocampus. This phenomenon explains how odors can evoke an emotionally charged memory just after exposure. At the same time, projections to the hypothalamus integrate olfactory input with autonomic and endocrine responses, while connections with the reticular formation influence arousal and vigilance levels [130,131].

### 4.3. Sedative, Relaxing, and Harmonizing Effects of YYEO

In addition to its cognitive and mood-related effects, YYEO has been widely associated with sedative and relaxing properties, although the strength and specificity of these effects remain subject to debate. Experimental studies have shown that inhalation of YYEO aroma can lead to measurable changes in physiological parameters, including reductions in heart rate and blood pressure, which are indicative of decreased sympathetic activity and a shift toward parasympathetic dominance. These changes are typically interpreted as markers of a sedative or anxiolytic response [2]. In addition, increased calmness and reduced tension were often reported in subjective reports, which aligns with the physiological findings. However, this effect is not purely sedative in a classical sense, and it has been called a “harmonizing” effect in some literature, characterized by a simultaneous reduction in physiological arousal and maintenance (or even slight enhancement) of attentional states. This suggests a more complex modulation of the autonomic nervous system rather than a simple suppression of central nervous system activity [132].

### 4.4. Antimicrobial Properties of YYEO in Textiles

The increasing problem of antimicrobial resistance, especially in bacteria, has made it necessary to look for new types of antibacterial finishes for textiles. Traditional antimicrobial agents are still widely used, but they can be toxic and harmful to the environment, which is why more attention is now being given to natural alternatives such as essential oils. The antibacterial effect of YYEO is usually explained by its interaction with the bacterial cell membrane. Since the oil is hydrophobic, it can penetrate lipid structures and disturb the integrity of the membrane, leading to increased permeability and leakage of intracellular components, which eventually causes the death of the cell [133]. As YYEO is volatile, it is important to develop a method or use some polymers to make it long-lasting on substrates. For instance, chitosan is widely used as a proper delivery system, utilizing a microencapsulation approach, leading to a long-lasting antibacterial effect of active substances and improving the oil delivery [134]. More advanced approaches involve nanotechnology, such as the use of silver nanoparticles synthesized with YYEO. In these systems, the oil not only acts as an antimicrobial agent but also helps in forming nanoparticles that show enhanced antibacterial activity. In this case, a well diffusion assay showed an inhibition zone of 27 mm for YYEO-capped silver nanoparticles against *E. coli* and 25 mm against *S. aureus*. The synergistic effect of the presence of silver nanoparticles and biomolecules of YYEO, which disrupt the permeability barrier of the microbial membrane and inhibit cellular respiration were reported to be the main reason for antibacterial activity [135]. Another study evaluated the different formulations of *C. odorata*, including hydroethanolic extract, oil, and nanoemulgel. The *C. odorata* nanoemulgel showed higher antibacterial activity, with inhibition zones reaching 21.32 mm against *E. coli* and 23.41 mm against *S. aureus* at 50 µg/mL. Remarkably, at this 5-fold lower concentration, the nanoemulgel showed larger inhibition zones than the reference antibiotic ciprofloxacin, which was 18.65 mm and 19.76 mm at 250 µg/mL, respectively [10]. Even though the results are promising, there are still some limitations that need to be taken into account. Different parameters, such as essential oil concentration and higher doses, may lead to cytotoxic effects and affect the effectiveness of YYEO, which means that careful formulation is necessary. In addition, the variability in the chemical composition of essential oils can make it difficult to obtain consistent results [12]. YYEO-based textile finishes seem to be a good alternative to synthetic antimicrobial agents, but more research is needed to improve their durability, safety, and reproducibility before they can be widely used in industrial applications.

### 4.5. Antioxidant Properties of YYEO

Biological systems and material stability can be affected by oxidative stress, especially in conditions where textiles are exposed to microorganisms, UV radiation, or reactive oxygen species (ROS). Because of this, antioxidant compounds are becoming increasingly important in the development of functional textiles. Natural antioxidants are often preferred over synthetic ones due to lower toxicity and better compatibility with biological systems. Experimental studies confirm that YYEO exhibits measurable antioxidant activity. In ABTS (2,2′-azino-bis(3-ethylbenzothiazoline-6-sulfonic acid) assays, YYEO showed an IC_50_ (Inhibitory Concentration 50%) value of approximately 21.89 µg/mL, indicating moderate radical scavenging ability compared to standard antioxidants. This activity is linked to the total phenolic content, which has been reported at around 9.67 mg GAE/g for the essential oil, suggesting that phenolic concentration plays a key role in antioxidant activity [136]. Similar observations have been reported in other studies, where extracts of YYEO demonstrated antioxidant activity in DPPH (2,2-diphenyl-1-picrylhydrazyl) assays. Results estimated the antioxidant activity to be 120.44 μg/mL (IC_50_). Chemical compounds of the essential oil such as carbohydrates, flavonoids, alkaloids, tannins, and phenolic compounds were reported to have antioxidant properties [137]. Beyond direct radical scavenging, YYEO may also influence endogenous antioxidant systems. In biological models, treatment with *C. odorata* extracts resulted in reduced levels of ROS and lipid peroxidation markers such as TBARS (Thiobarbituric Acid Reactive Substances), along with increased activity of antioxidant enzymes like superoxide dismutase (SOD) and glutathione (GSH). This suggests that its activity is not limited to chemical interactions, but also involves modulation of biological defense mechanisms [138]. As discussed, different studies employed different assays, concentrations, extraction methods, and reference standards. For example, DPPH, ABTS, FRAP, and cellular oxidative-stress models. It is worth mentioning that these approaches cannot be directly compared using a single numerical scale, as they measure different aspects and behaviors of antioxidant materials. Furthermore, the relative abundance of oxygenated monoterpenes and sesquiterpenes, which varies according to geographical origin, plant material, developmental stage, extraction conditions, and other constituents of the extracts, may strongly affect the antioxidant activity. Therefore, the reported antioxidant activity of YYEO should be interpreted carefully.

### 4.6. Anti-Inflammatory Properties of YYEO

YYEO has also been reported to show anti-inflammatory activity, although most of the available data come from in vitro and animal studies rather than clinical research [139,140,141]. The proposed mechanism is mainly related to the modulation of early stages of the inflammatory response, especially those leading to activation of leukocytes and their migration. Experimental results indicate that YYEO can reduce neutrophil chemotaxis as well as phagocytic activity, which suggests control of immune cell activation at the initial phase of inflammation. Moreover, treatment with YYEO led to a decrease in total leukocyte recruitment and nitric oxide production, pointing to a reduction in pro-inflammatory signaling [142]. From a molecular perspective, these effects may be connected to the downregulation of pathways involved in inflammation, including MAPKs, NOS2, and NF-κB-related markers, which have been associated with neuroinflammatory processes [143]. It is also worth noting that antioxidant activity may contribute to the observed effects, as reducing oxidative stress can indirectly limit inflammatory responses [144]. Similar to other essential oils, it is difficult to attribute these effects to a single mechanism, since they most likely result from combined with different compounds. Therefore, inflammatory effects may be the result of linalool, which contributes to topical anti-inflammatory and analgesic activities by restraining COX-2 protein expression in inflammatory tissues and decreasing oxidative stress [145]. In another study, Germacrene D decreased inflammation by preventing the release of inflammatory mediators by disabling the enzymes that cause inflammation. The most important mechanism of Germacrene D is the inhibition of prostaglandin synthesis. Prostaglandins are compounds affecting the inflammatory response and pain generation. This inhibition both alleviates inflammation and helps reduce pain [146].

### 4.7. Spermatotoxic Effect of YYEO

The antifertility and spermatotoxic potential of YYEO has attracted interest in male contraceptive research, with studies identifying a specific 52 kDa protein fraction extracted from its root bark as the main bioactive agent [147,148]. Oral administration of a 50% ethanolic extract of the root bark to male albino models over 60 days results in a significant decrease in epididymal sperm count, motility, and overall viability, leading to a significant drop in the male fertility index. Mechanistically, this spermatotoxic action operates by disrupting testicular steroidogenesis; the extract significantly inhibits 3-hydroxy-3-methylglutaryl-coenzyme A (HMG-CoA) reductase, leading to an accumulation of testicular cholesterol and an instantaneous downregulation of key steroidogenic enzymes, specifically 3β-hydroxysteroid dehydrogenase (3β-HSD) and glucose-6-phosphate dehydrogenase (G6PD) [1,147]. The resulting androgen deficiency decreases the level of serum testosterone below the critical threshold required to maintain spermatogenesis, inducing severe morphological abnormalities during epididymal sperm maturation. Importantly, YYEO shows a highly beneficial therapeutic profile in comparison to the classical antifertility benchmark gossypol. Although both agents achieve a comparable suppression of fertility, the spermatotoxic damage induced by YYEO is completely reversible, with epididymal sperm parameters recovering full physiological motility and functionality within the withdrawal period of 15 days [144].

### 4.8. Future Trends and Emerging Frontiers

The future of using YYEO in protective clothing may be achieved by matching it with discoveries in biology, nanotechnology, and smart clothing. These advanced methods may finally address the YYEO’s biggest weaknesses, which are its high volatility, its tendency to wash out easily, and loss of active groups during storage and processing. Therefore, further studies should focus on stabilizing strategies such as microencapsulation, nanoencapsulation, and the incorporation of the oil into polymeric or other carrier systems, with special attention to washing durability, retention of bioactive compounds, and release kinetics.

Another important research priority may be standardization of YYEO. Future studies should establish a clear relationship between chemical composition and biological performance using standardized analytical and biological protocols because the chemical composition of the YYEO substantially depends on geographical origin, growth conditions, extraction techniques, and distillation fraction. Such studies would help identify the constituents or compositional profiles associated with antimicrobial, insecticidal, repellent, antioxidant, or other functional effects.

For textile applications, proof-of-concept demonstrations are not enough on their own. Research should move beyond and evaluate practical performance under realistic conditions. Important parameters, including resistance to repeated laundering, controlled release, long-term storage stability, mechanical and sensory properties of the treated materials, skin compatibility, and potential cytotoxicity, should be considered. Comparisons with conventional synthetic finishes and commercially available repellent systems would also be valuable for assessing practical feasibility.

Stimuli-responsive release systems may be a potential long-term approach, particularly when they are based on proven responsive carrier materials. However, their application to Ylang-Ylang essential oil remains largely unexplored and should first be investigated through controlled studies of trigger mechanisms, release kinetics, safety, and biological performance.

At the production level, further optimization of microwave-assisted distillation, supercritical fluid extraction, and other lower-impact extraction technologies should take into account not only extraction yield and chemical composition but also energy consumption, water use, scalability, and economic viability. In addition, the valorization of post-extraction biomass may support more circular production systems, although the biological activity, safety, and processing requirements of these residual materials should be assessed prior to actual applications.

### 4.9. Conclusions

This review summarizes current knowledge on YYEO, emphasizing the relationships between extraction technology, chemical composition, biological properties, and potential applications. The available evidence demonstrates that extraction conditions have a substantial influence on both process efficiency and the chemical profile of the resulting oil. Conventional hydrodistillation is simple and established, making it industrially relevant; however, its long processing time, high energy and water requirements, and potential degradation of thermosensitive compounds represent important limitations. In comparison, microwave-assisted processing can reduce extraction time from approximately 19 h to about 40 min while maintaining a high proportion of aroma-relevant light oxygenated compounds. Supercritical fluid extraction also offers advantages in terms of reduced thermal exposure and product purity, although higher equipment costs and operational requirements may limit its broader application.

The reviewed studies further indicate that YYEO possesses antioxidant, antimicrobial, anti-inflammatory, insecticidal, repellent, and mood-related properties, although the strength of evidence varies substantially among these applications. Particularly promising results have been reported for functional textiles, including mosquito-repellent fabrics containing microencapsulated YYEO. Nevertheless, many biological findings are still based on in vitro or animal studies, and evidence derived from non-volatile *C. odorata* extracts or isolated constituents should not be directly extrapolated to the complete essential oil. Future research should therefore prioritize standardized YYEO composition, directly comparable extraction conditions, safety assessment, long-term stability, and well-designed biological and application-oriented studies.

## Figures and Tables

**Table 1:** Integrated Profile of *C. odorata* [1].

Biological Aspect	Categorization and Descriptive Data
Taxonomic Position	Member of the Annonaceae family (custard-apple group) within the order Magnoliales. Classified under the division Magnoliophyta as a vascular, seed-bearing dicotyledon.
Scientific Nomenclature	*Cananga odorata* (Lam.) Hook. f. & Thomson. Primary synonyms include Uvaria odorata Lam. and *Canangium odoratum* (Lam.) Baill. ex King.
Botanical Characteristics	A perennial tropical evergreen reaching heights of approximately 15 m. Features drooping branches and ovate–oblong leaves (9–21 cm) with a distinct wavy margin.
Floral Morphology	Highly fragrant, hanging axillary clusters of 4–12 flowers. Petals are 4–6 cm long, transitioning from yellowish-green (immature) to a rich yellow at full maturity.
Key Phytochemicals	Rich in monoterpenes (Linalool, Geraniol), sesquiterpenes (β-Caryophyllene, Germacrene D), and phenylpropanoids (Benzyl acetate, Methyl benzoate).
Industrial Classification	Essential oils are graded by distillation time: Extra (highest volatiles), First, Second, and Third (highest sesquiterpene content).

**Table 2:** Reported applications of microwave-assisted extraction for YYEO and selected aromatic plants included for methodological context.

Extraction Method	Extracted Essential Oil	Reported Yield (Original Unit and Basis)	Density (g/mL)	Water/Material (V/m) or Solvent/Material Ratio (V/m)	Plant Part/State	Sample Mass (g)	Time/Energy	Ref.
Microwave-Assisted distillation	YYEO	2.7%	0.976	1:1	Flower/Fresh	200	40 min/750 W	[8]
Microwave-Assisted Extraction	YYEO	27.6%	NA *	19:1	Flower/Dried	5	45 min/375 W	[56]
Microwave-Assisted Extraction	Citronella, Patchouli	2.11% for Citronella and 5.4% for Patchouli	NA	NA	Leaves/ Fresh	250	3 h/400 W, 500 W, 600 W and 700 W (for Citronella) 6 h/600 W, 700 W, 800 W and 900 W (for Patchouli)	[57]
Microwave-Assisted Extraction	Rose Hybrid Tea	0.012%	1.096	3:1	Flower	NA	15 min/300 W, 25 min/300 W, 35 min/300 W, 15 min/400 W, 25 min/400 W, 35 min/400 W, 15 min/500 W, 25 min/500 W, 35 min/500 W,	[58]
Microwave-Assisted Extraction	YYEO	NA	NA	2:1	Flower/Fresh	200	180 min/200 W, 400 W, and 600 W	[59]
Microwave-Assisted Extraction	*Ocimum basilicum* L, *Coriandrum sativum* L.	0.527 ± 0.025% (*Ocimum basilicum*) 0.693 ± 0.026% (*Coriandrum sativum*)	0.959 ± 0.021 (*Ocimum basilicum*) 0.933 ± 0.015 (*Coriandrum sativum*)	Solvent-free methods are used	Leaves/dried, Seeds/dried	400 g	70 min/900 ± 10 W	[60]
Microwave-Assisted Extraction	*Ocimum basilicum* L.	1.7 ± 0.1%	NA	10:1	Aerial parts/dried	20	15 min/500 W	[61]
Microwave-Assisted Hydrodistillation	*Cymbopogon flexuosus*	1.89%	NA	6.25:1	Leaves/Fresh	80	50 min/700 W	[62]
Solvent Free Microwave-assisted distillation	YYEO	0.956 ± 0.06%	0.988 ± 0.002	Solvent-free methods are used	Flower/Fresh	100	60 min/350 W	[12]
Microwave-assisted steam distillation	*Paeonia suffruticosa Andrews*	0.66 ± 0.03 mg/g **	NA	1.7:1	Flower/Dried	NA	30 min/540 W	[63]
Microwave-assisted extraction	*Mentha arvensis* L.	0.0832 mL/g	NA	1:1, 1:1.5, 1:2 and 1:2.5	Leaves/Dried	200	10–25 min/230 W, 380 W, 540 W, and 700 W	[64]

* Not Available; ** Yield values are reported as originally defined in the cited studies. Because the plant material, moisture basis, extraction scale, and yield definitions differ among studies, the reported values should not be directly compared or interpreted as a universal ranking of extraction efficiency.

**Table 3:** Application of supercritical fluid extraction to YYEO and selected aromatic plant materials.

Essential Oil	Temperature (°C)	Pressure (MPa)	Flow Rate	Extraction Time (min)	Particle Size (mm)	Yield (%)	Ref.
Limonene	Between 20 and 50	Between 8 and 28	0.5 to 3.5 kg h^−1^	NA *	0.1 to 10	99.5	[72]
Citrus reticulata	43	29.7	10 g/min	120	<0.5	1.57	[73]
Oregano, lavender, sage, anise, clove	50	20	3 g/min	120	NA	Oregano (3.04), lavender (5.11), sage (2.31), anise (2.91), clove (18.44)	[74]
Mahua	40	10	30 g/min	120	NA	2.074	[75]
Hetian Rose	Between 35 and 45	Between 30 and 40	10 L/h	120	0.8	8.99	[76]
Rosemary	45 and 65	Between 15 and 25	NA	150	Between 0.9 and 0.15	1.87	[77]
Coriander seed	60	25	500 mL/min	90	NA	14,7	[78]
Citron	35	10	NA	30	NA	7.6	[79]
*Origanum vulgare* L.	54	21.7	3 L/h	120	NA	1.136	[80]
Camphor Tree	45	25	NA	150	NA	4.63	[81]
Peppermint	40	40	0.3 kg/h	30	0.4	3.68	[22]
Ylang-Ylang	35	12	3 L/min	210	NA	0.965	[82]
Tangerine peel	45	14	NA	147	0.2–1.0	1.34	[83]

* Not available.

**Table 4:** Qualitative comparison of conventional and advanced extraction technologies.

Extraction Method	Mechanism	Time Requirement	Solvent Use	Product Quality	Environmental Impact
Hydrodistillation (HD)	Thermal conduction/Steam	High (Up to 20–24 h)	Large volumes of water	Risk of thermal degradation	High energy/water footprint
Microwave-Assisted (MAD)	Selective dielectric heating	Low (approx. 40 min)	Internal plant moisture	High retention of top notes	Significant energy reduction
Supercritical Fluid (SFE)	Diffusivity and Solubility	Medium	CO_2_ (Recyclable)	Solvent-free, high purity	Low, no toxic residues
Ultrasound-Assisted (UAE)	Acoustic cavitation	Low to Medium	Minimal water/ethanol	High yield of bioactives	Process intensification

**Table 5:** Important characteristics that should be considered for textile applications.

Category	Desired Characteristics
Safety & Bioavailability	Low toxicity, minimal skin permeation, non-irritating.
Efficacy	Broad-spectrum action, long-lasting, water/sweat resistant.
User Experience	Pleasant/neutral scent, non-greasy, stain-resistant on fabrics, low cost.
Material Stability	Chemically stable, abrasion-resistant, non-reactive to plastics/synthetics.

## Data Availability

No new data were created or analyzed in this study. Data sharing is not applicable to this article.

## Abbreviations

YYEO	Ylang-Ylang essential oil
*C. odorata*	*Cananga odorata* (Lam.) Hook.f. & Thomson
HD	Hydrodistillation
WHD	Wood-heated distillation
MAD	Microwave-assisted distillation
MAE	Microwave-assisted extraction
SFE	Supercritical fluid extraction
UAE	Ultrasound-assisted extraction
LOCs	Light oxygenated compounds
LD	Laboratory-scale distillation
BIC	Broken and Intact Cell
CER	Constant extraction rate
FER	Falling extraction rate
DC	Diffusion-controlled
NADES	Natural deep eutectic solvents
VOC	Volatile organic compound
GRAS	Generally Recognized as Safe
DENV	Dengue virus
ZIKAV	Zika virus
GBS	Guillain–Barré syndrome
WHO	World Health Organization
DEET	N,N-diethyl-3-methylbenzamide
MIC	Minimum inhibitory concentration
ROS	Reactive oxygen species
ABTS	2,2′-Azino-bis(3-ethylbenzothiazoline-6-sulfonic acid)
IC_50_	Inhibitory concentration 50%
DPPH	2,2-Diphenyl-1-picrylhydrazyl
TBARS	Thiobarbituric acid reactive substances
SOD	Superoxide dismutase
GSH	Glutathione
MAPKs	Mitogen-activated protein kinases
NOS2	Nitric oxide synthase 2
NF-κB	Nuclear factor kappa B
COX-2	Cyclooxygenase-2
HMG-CoA	3-Hydroxy-3-methylglutaryl-coenzyme A
3β-HSD	3β-Hydroxysteroid dehydrogenase
G6PD	Glucose-6-phosphate dehydrogenase
